# On As(III) Adsorption Characteristics of Innovative Magnetite Graphene Oxide Chitosan Microsphere

**DOI:** 10.3390/ma15207156

**Published:** 2022-10-14

**Authors:** Huimei Shan, Yunquan Liu, Chunya Zeng, Sanxi Peng, Hongbin Zhan

**Affiliations:** 1 Guangxi Key Laboratory of Environmental Pollution Control Theory and Technology, Guilin University of Technology, Guilin 541004, China; 2 College of Environmental Science and Engineering, Guilin University of Technology, Guilin 541004, China; 3 Department of Geology & Geophysics, Texas A&M University, College Station, TX 77843, USA

**Keywords:** chitosan, graphene oxide, magnetite, As(III) removal

## Abstract

A magnetite graphene oxide chitosan (MGOCS) composite microsphere was specifically prepared to efficiently adsorb As(III) from aqueous solutions. The characterization analysis of BET, XRD, VSM, TG, FTIR, XPS, and SEM-EDS was used to identify the characteristics and adsorption mechanism. Batch experiments were carried out to determine the effects of the operational parameters and to evaluate the adsorption kinetic and equilibrium isotherm. The results show that the MGOCS composite microsphere with a particle size of about 1.5 mm can be prepared by a straightforward method of dropping FeCl_2_, graphene oxide (GO), and chitosan (CS) mixtures into NaOH solutions and then drying the mixed solutions at 45 °C. The produced MGOCS had a strong thermal stability with a mass loss of <30% below 620 °C. The specific surface area and saturation magnetization of the produced MGOCS was 66.85 m^2^/g and 24.35 emu/g, respectively. The As(III) adsorption capacity (*Q_e_*) and removal efficiency (*R_e_*) was only 0.25 mg/g and 5.81% for GOCS, respectively. After 0.08 mol of Fe_3_O_4_ modification, more than 53% of As(III) was efficiently removed by the formed MGOCS from aqueous solutions over a wide pH range of 5–10, and this was almost unaffected by temperature. The coexisting ion of PO_4_^3−^ decreased *Q_e_* from 3.81 mg/g to 1.32 mg/g, but Mn^2+^ increased *Q_e_* from 3.50 mg/g to 4.19 mg/g. The As(III) adsorption fitted the best to the pseudo-second-order kinetic model, and the maximum *Q_e_* was 20.72 mg/g as fitted by the Sips model. After four times regeneration, the *R_e_* value of As(III) slightly decreased from 76.2% to 73.8%, and no secondary pollution of Fe happened. Chemisorption is the major mechanism for As(III) adsorption, and As(III) was adsorbed on the surface and interior of the MGOCS, while the adsorbed As(III) was partially oxidized to As(V) accompanied by the reduction of Fe(III) to Fe(II). The produced As(V) was further adsorbed through ligand exchange (by forming Fe–O–As complexes) and electrostatic attraction, enhancing the As(III) removal. As an easily prepared and environmental-friendly composite, MGOCS not only greatly adsorbs As(III) but also effectively removes Cr(VI) and As(V) (*R_e_* > 60%) and other metals, showing a great advantage in the treatment of heavy metal-contaminated water.

## 1. Introduction

Arsenic pollution in the environment has attracted increasing attention worldwide [1,2]. Long-term exposure to As-containing water, which is more than the limit of 10 μg/L in drinking water suggested by the World Health Organization (WHO), can cause bladder, skin cancer, and cardiovascular disease [3]. The main species of As in water usually include As(III) (as AsO_3_^3−^) and As(V) (as AsO_4_^3−^), and the former shows higher toxicity and stronger mobility [4]. Therefore, it is more difficult to efficiently remove As(III).

Several methods have been developed for As(III) removal, such as ion exchange [5], biological methods [6,7], coprecipitation [8], adsorption [9], etc. Among them, adsorption is recognized as one of the most promising methods because it has the advantages of simple operation, low cost, good selectivity and sensitivity, high efficiency, etc. In recent decades, composite materials have generated growing attention among the environmental material communities for their diverse anion removal capability based on their specific functionality and surface area [10,11,12], such as multifunctional resins/fibers [13], biochar-based materials [14], multiple metals (Fe, Mn, Zr, etc.) or their oxides composites [15,16], etc.

Chitosan (CS) is a natural biodegradable polymer material with a large number of amino and hydroxyl groups that can be ligated with heavy metal ions through a chelation reaction and has been recognized as an effective new adsorbent [12,17,18]. However, it is less stable and easily dissolvable in acidic media. Therefore, it is necessary to overcome these problems with an appropriate modification when it is applied to acidic wastewater treatment. Recently, some researchers found that by combining graphene oxide (GO) with CS properly and/or simultaneously introducing some metals into them to form composites, the performance can be improved, such as the stability, reusability, the number and variety of functional groups related to pollutant removal, etc., [19,20,21] as GO has a huge specific surface area (theoretically ~2600 m^2^/g) and is rich in oxygen-containing functional groups such as hydroxyl, carboxyl, and epoxy groups [22,23]. These characteristics allow GO to easily adsorb heavy metal ions and be recognized as a type of nanosorbent material widely applied in wastewater treatment. Meanwhile, the introduction of iron-based materials into GO and/or CS has also attracted interest because the iron-based adsorbents show advantages of high efficiency in heavy metal remediation, environmental friendliness, and abundance on earth. Such iron-based materials include α–Fe_2_O_3_, α–FeOOH, Fe^0^, Fe_3_O_4_, etc. Among them, Fe_3_O_4_ decoration has been given the most attention because it not only enhances the adsorption but also makes the composite materials more easily separatable from water using an external magnetic field without centrifugation or filtration [19,24,25,26,27]. For example, the Fe_3_O_4_/GO/CS composite has been proven to be an excellent nanocomposite showing higher adsorption capacities for Cr(VI) [28,29,30], Pb(II) [31], methylene blue [32], and Disperse Blue 367 [33], etc., and shows better regeneration than most other nanocomposites. Compared to those studies, Fe_3_O_4_/GO/CS composite application for arsenic adsorption has not attracted much attention [19] because arsenic speciation is different under varying pH and redox conditions, leading to the adsorption of arsenic being rather complex. Meanwhile, although the decoration of a magnetite can improve the adsorption of the composite, the stability of Fe_3_O_4_/GO/CS and the potential of Fe secondary pollution caused by magnetite dissolution are rarely reported. Furthermore, most previous studies usually use Fe(III) or a certain ratio of Fe(III) and Fe(II) to react at a high temperature (~200 °C) to generate Fe_3_O_4_ and magnetic decoration for the composite [23,31,33,34], and the processes are complicated. Therefore, developing a straightforward method for preparing a magnetite graphene oxide chitosan composite and evaluating its stability, adsorption performance, and mechanism for arsenic is of significant interest.

This study aims to develop an easily operational method to synthesize a magnetite graphene oxide chitosan (MGOCS) composite and test its performance with regards to As(III) adsorption. Batch experiments were conducted to determine the effects of operational parameters on the As(III) removal, including Fe-loading mass, mass and volume ratio (*m*/*v*), initial As(III) concentration, pH, coexisting ions, temperatures, etc., and the characteristics of the kinetic and isothermal adsorption of As(III). Combined with the characterization analysis of Brunauer–Emmett–Teller (BET), X-ray powder diffraction (XRD), a vibrating sampling magnetometer (VSM), a thermogravimetric analyzer (TG), Fourier transform infrared spectroscopy (FTIR), an X-ray photoelectron spectroscopy (XPS), and scanning electron microscopy with energy dispersive spectrometer (SEM-EDS), the physical and chemical characteristics of MGOCS and its adsorption mechanism of As(III) were further determined.

## 2. Materials and Methods

### 2.1. Materials

CS (deacetylation ≥90%) was purchased from Lanji Technology Development Co., Ltd. (Shanghai, China). GO (purity >90 wt%, a thickness of 3.4–7.0 nm, 5–10 layers, a diameter of 10–50 μm, and a specific surface area of 100–300 m^2^/g) was purchased from Tanfeng Graphene Technology Co., Ltd. (Suzhou, China). Glutaraldehyde with a purity of 50% was purchased from Aladdin Reagent Co., Ltd. (Shanghai, China). Sodium arsenite (as As(III)) was purchased from Best Reagent Co., Ltd. (Chengdu, China). Other reagents and chemicals including HCl, FeCl_2_·4H_2_O, NaOH, anhydrous ethanol, acetic acid, and methanol were analytical grade and purchased from Xilong Science Co., Ltd. (Shanghai, China). Deionized water was prepared using a Milli-Q water system (Millipore, Boston, MA, USA).

### 2.2. MGOCS Preparation

MGOCS composite microspheres were prepared by a novel and easily operational method using the following steps. Firstly, 0.8 g of GO was added into 200 mL 1.5% acetic acid solution and ultrasonically stirred for 60 min. Then, 4.0 g of CS was added to this solution and ultrasonically stirred for 30 min. After that, 0.08 mol FeCl_2_·4H_2_O (this optimum content was determined from the batch experiments in Section 2.3) was added into this mixture and ultrasonically stirred for about 15 min, and the obtained mixture solutions containing Fe, GO and CS were dropped into 5% NaOH solutions through a 10 mL syringe with a needle at a rate of 1 drop per second. After standing for 24 h at room temperature, the composite microspheres were formed in the solutions and precipitated on the bottom. These microspheres were filtered and repeatedly washed using deionized water until the pH was stable. Finally, these microspheres were put into a 5% glutaraldehyde-methanol solution to stir for 2 h, then the solid microspheres were washed using deionized water and ethanol until the pH of the solutions was stable. The obtained product after being dried at 45 °C was named MGOCS (Figure S1, Supplementary Materials), which had a particle size of about 1.5 mm.

### 2.3. Batch Experiments

Batch experiments were conducted to determine the adsorption characteristics of MGOCS composite microspheres for As(III) removal from aqueous solution and the effects of operational parameters including Fe content of 0.2–1.0 mol, initial As(III) concentrations of 2–200 mg/L, mass and volume ratio (*m*/*v*) of 0.2–3.0 g/L, pH of 3–11, coexisting ions, and temperature (*T*) of 25 °C, 35 °C, and 45 °C. The pH was adjusted using 0.1 M HCl and 0.1 M NaOH. All the experiments were carried out in triplicate at a shaking rate of 180 rpm under optimal conditions unless otherwise stated. All the solutions before and after the reaction were collected for As(III) concentration measurement.

The equilibrium adsorption capacity (*Q_e_*, mg/g) and removal efficiency of As(III) (*R_e_*, %) was calculated according to Equations (1) and (2), respectively.

(1) $$R_{e}=\frac{\left(C_{0}-C_{e}\right)}{C_{0}}\times 100\;$$

(2) $$Q_{e}=\frac{\left(C_{0}-C_{e}\right)}{m}\times V$$

where *C*_0_ and *C_e_* (mg/L) are the initial and equilibrium concentrations of As(III), respectively, *V* (L) is the volume of an aqueous solution, and *m* (g) is the mass of the adsorbent.

### 2.4. Kinetic and Isotherm Adsorption

Adsorption kinetic results were used to fit with the pseudo-first-order and pseudo-second-order kinetic models, expressed as the following Equations (3) and (4), respectively:

(3) $$Q_{t}=Q_{e}\left(1-e^{-k_{1}t}\right)$$

(4) $$Q_{t}=\frac{Q_{e}^{2}k_{2}t}{1+Q_{e}k_{2}t}$$

where *Q_e_* (mg/g) and *Q_t_* (mg/g) are the adsorption capacities of As(III) at equilibrium time and *t*, respectively; and *k*_1_ (min^−1^) and *k*_2_ (mg·(g·min)^−1^) are the first-order and the second-order rate constants, respectively. The adsorption isotherm was used to fit the Langmuir, Freundlich, and Sips models. Their equations are, respectively, as follows:

(5) $$\mathrm{Langmuir}\;\mathrm{model}:\;Q_{e}=\frac{Q_{m}K_{L}C_{e}}{1+K_{L}C_{e}}$$

(6) $$\mathrm{Freundlich}\;\mathrm{model}:\;Q_{e}=K_{f}C_{e}^{\frac{1}{n}}$$

(7) $$\mathrm{Sips}\;\mathrm{model}:\;Q_{e}=\frac{Q_{m}K_{s}C_{e}{}^{1/m}}{1+K_{s}C_{e}{}^{1/m}}$$

where *Q_e_* is the adsorption capacity (mg/g); *C_e_* is the equilibrium concentration of As(III) (mg/L); *Q_m_* is the maximum adsorption capacity of As(III) (mg/g); *K_L_* is the Langmuir adsorption equilibrium constant, which is related to the strength of the adsorption interaction; *K_f_* and 1/*n* are the adsorption equilibrium constant and the adsorption strength constant of the Freundlich equation, respectively, and a smaller 1/*n* means a better adsorption capacity; *K_s_* is the equilibrium constant for heterogeneous solids; and *m* is a heterogeneous parameter.

### 2.5. Regeneration Experiment

After each adsorption experiment, MGOCS composite microspheres were regenerated by being immersed into 0.1 M NaOH for 24 h at 25 °C and then washed using deionized water until the pH of solutions was stable at about 7. After that, the microspheres were dried at 45 °C in a blast dryer and used for As(III) adsorption experiments again. Such regeneration experiments were repeated four times to determine the reusability of MGOCS for As(III) removal.

### 2.6. Analytical Techniques

As(III) concentration of solutions was measured using an inductively coupled plasma emission spectroscopy (ICP-OES, Optima 7000DV, Platinum Elmer Instruments, Inc., Waltham, MA, USA), and the relative standard deviation was less than 5%. The surface area and pore size distribution were determined through the N_2_ adsorption–desorption isotherms using an autosorb-iQ analyzer (ASAP 2020, Micromeritics, Norcross, GA, USA). The crystal structure was determined by the X’Pert3 Powder type multifunctional X-ray diffraction (XRD) analyzer (Panaco, Cu target, λ = 1.54056 Å, Almelo, The Netherlands). The test scan step was 0.0263° at a speed of 0.6565°/s, and the scan angle was 5°–80°. The magnetization curve was measured at room temperature using a vibrating sample magnetometer (Lake Shore 7404). The thermal stability was analyzed using a thermogravimetric analyzer (TGA 209F3, NETZSCH, Germany), and the measurement conditions were the temperature range of 20–700 °C, the heating rate of 10 °C/min, and nitrogen protection at the flow rate of 30 mL/min. Functional groups and structures were determined by a Fourier transform infrared spectroscopy (FTIR) analyzer (IS10, Thermo Fisher, Waltham, MA, USA) and an X-ray photoelectron spectroscopy (XPS) analyzer (WISDOM9600, China), respectively. The surface morphology and elemental compositions were characterized using scanning electron microscopy (SEM) with energy dispersive X-ray spectroscopy (EDS) (JSM7900F, JEOL, Japan). The zeta potential (pH*_zpc_*) of MGOCS in an aqueous dispersion was measured by the zetasizer nano analyzer (Malvern, Nano zs90, Melvin, UK).

## 3. Results and Discussion

### 3.1. Characterization Analysis

#### 3.1.1. BET Analysis

**Figure 1:**
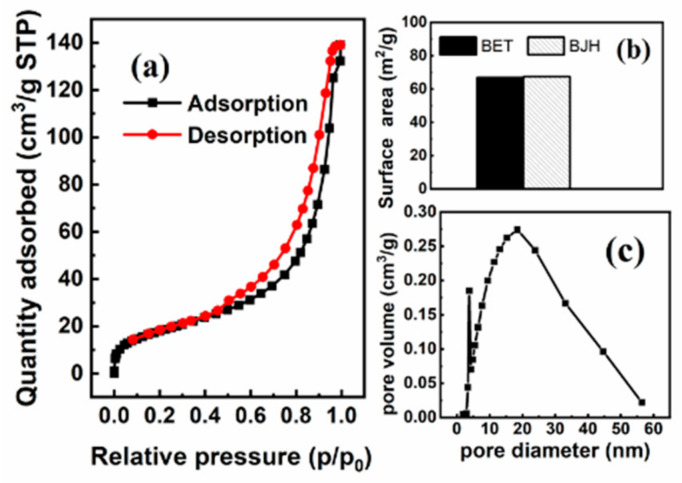
BET analysis of the MGOCS: (**a**) adsorption/desorption isotherm plots, (**b**) bar graphs of the BET and BJH surface areas, and (**c**) BJH plots of the pore size distribution.

Figure 1 shows the surface area and pore size distribution of MGOCS from the N_2_ adsorption–desorption isothermal curve. There is an obvious desorption hysteresis for MGOCS exhibiting a type IV adsorption isotherm as referred to in the IUPAC classification, indicating that it is a type of mesoporous material. Compared to the GOCS composite (Figure S2, Supplementary Materials), the total pore volume and the surface area of MGCOS are greatly improved after iron modification. The total pore volume and the average pore size of MGOCS are 0.22 cm^3^/g and 12.88 nm, respectively, which is higher than that of the GOCS composite (0.23 × 10^−3^ cm^3^/g and 4.18 nm, respectively). The surface areas of MGOCS are 66.85 m^2^/g and 67.39 m^2^/g, calculated by the Brunauer–Emmett–Teller (BET) and Barrett–Joyner–Halenda (BJH) methods, respectively, which are larger than that of the GOCS composite (4.22 m^2^/g).

#### 3.1.2. XRD Analysis

**Figure 2:**
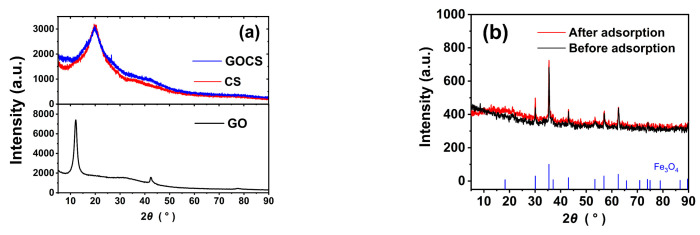
(**a**) XRD patterns of GO, CS, and GOCS; (**b**) MGOCS before and after adsorption of As(III).

Figure 2 shows the XRD patterns of the samples. GO has two prominent peaks at 2*θ* = 10.71° and 2*θ* = 42.35°, respectively. CS has a prominent peak at 2*θ* = 20.06°. After incorporating GO into CS, there is only one prominent peak at 2*θ* = 20.20°, indicating that GO and CS are successfully assembled. The main characteristic peaks of the MGOCS appear at 2*θ* = 30.14°, 35.58°, 43.20°, 57.08°, and 62.68°, similar to that of the standard Fe_3_O_4_ (ICDD PDF No.65-3107(2022)). In addition, the characteristic peaks are sharp, and no other peaks related to impurities are observed, indicating the pure magnetite phase of Fe_3_O_4_ in the composites [35]. After the MGOCS adsorption of As(III), the major characteristic peaks of Fe_3_O_4_ are slightly enhanced (red line, Figure 2b), indicating that Fe_3_O_4_ may be involved in the As(III) adsorption.

#### 3.1.3. VSM Analysis

**Figure 3:**
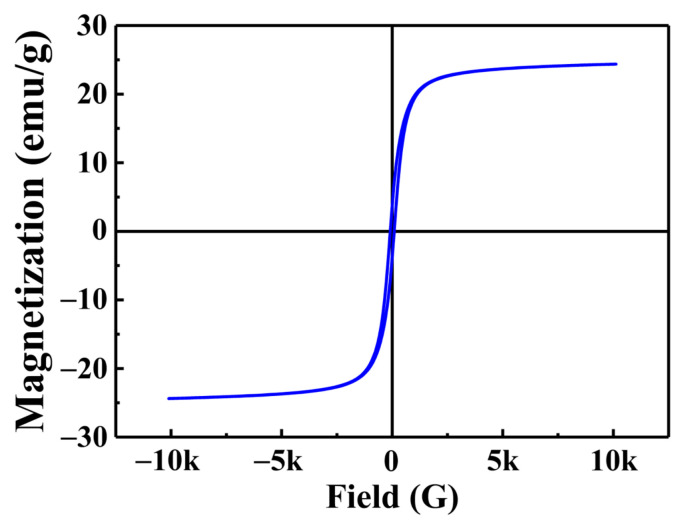
Magnetization curves of MGOCS.

Figure 3 shows the magnetic hysteresis curves of the MGOCS composite microspheres measured by the vibrating sampling magnetometer (VSM). It can be seen that there is no hysteresis, and both remanence and coercivity are zero, suggesting that the samples are superparamagnetic [36]. In addition, the saturation magnetization intensity of the MGOCS composite microsphere is 24.35 emu/g, meaning that the MGOCS composite microspheres can be easily separated from aqueous solutions using an external magnetic field [37].

#### 3.1.4. TG Analysis

**Figure 4:**
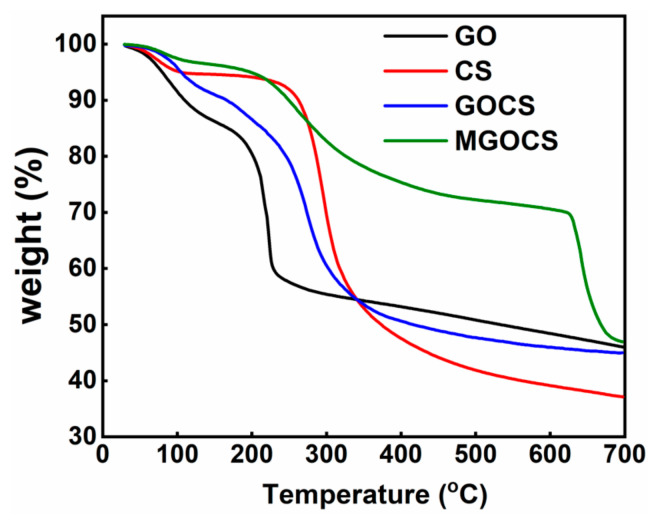
TG analysis of GO, CS, GOCS, and MGOCS.

The pyrolysis characteristics of GO, CS, GOCS, and MGOCS tested with a thermogravimetric analyzer (TG) are shown in Figure 4. It can be seen that at a temperature lower than 200 °C, the weight loss of GO is about 20%, which is mainly from its surface and bound water. When the temperature increases from 200 °C to 230 °C, there is a significant drop in the weight of GO from 80% to 60%, which is mainly caused by its unstable functional groups and carbon skeleton decomposition. When the temperature increases to 700 °C, the weight gradually decreases, and the final residual weight is about 46%. For CS, there is a small weight loss of less than 5% with the increasing temperature from 30 °C to 120 °C, mainly attributed to the loss of residual acetic acid and water molecules. When the temperature increases to 200 °C, the weight keeps stable at about 94%. After that, there is a great weight loss mainly caused by the thermal decomposition of the glycosidic bonds in the internal polysaccharides into fatty acids such as acetic acid and butyric acid [38], and at 700 °C, the residual weight is only about 37%. After the incorporation of GO into CS to obtain GOCS, with the increasing temperatures from 20 °C to 700 °C, the weight gradually decreases to 45%. Specifically, when the temperature is lower than 100 °C, the weight of GOCS is slightly larger than that of GO or CS at the same temperature, indicating that the interaction of GO and CS can improve the stability of a composite at a lower temperature. When the temperature ranged from 100 °C to 340 °C, the weight of GOCS is larger than that of GO and smaller than that of CS, but the result is the opposite at 340–700 °C. This means that the interaction of GO and CS can improve the thermal stability of GO at a medium temperature and enhance the thermal stability of CS at a higher temperature. This is consistent with the studies of Han et al. [39], Jadoon et al. [40], and Huo et al. [41]. For MGOCS, the thermal stability is greatly enhanced compared to individual GO and CS, and the GOCS composite. It was found that with increasing temperatures from 20 °C to 620 °C, the weight loss is much smaller for MGOCS (about 30%) than for GO, CS, and GOCS (50–60%). After that, there is a significant drop of weight to 47%, meaning that the rapid thermal decomposition of MGOCS mainly occurs at higher than 620 °C.

#### 3.1.5. FTIR Analysis

**Figure 5:**
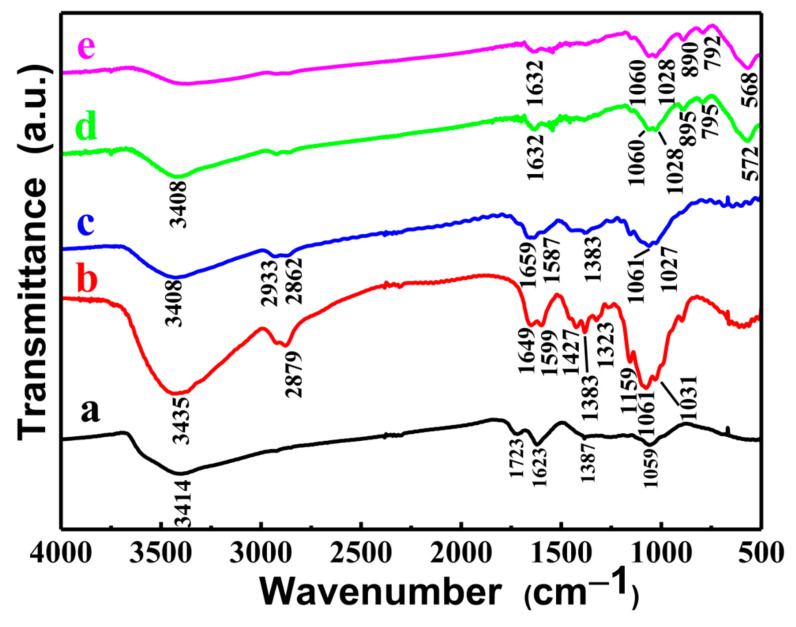
FTIR spectra of (a) GO, (b) CS, (c) GOCS, and MGOCS before (d) and after (e) As(III) adsorption.

Figure 5 shows the FTIR spectrums of GO, CS, GOCS, and the MGOCS composite microspheres. From the GO spectrum, the band at 3414 cm^−1^ is due to the O–H group, and the bands at 1723 cm^−1^, 1623 cm^−1^, and 1387 cm^−1^ are assigned to the C=O, C=C, and C–O vibrations, respectively, and the absorption band at 1059 cm^−1^ is corresponding to the C–O vibration of an epoxy group [42,43]. From the CS spectrum, the broadband at about 3435 cm^−1^ is attributed to the overlap of O−H and N−H stretching vibrations [44], and the band at 2879 cm^−1^ is caused by the C−H stretching vibration in methyl groups. The absorption bands at about 1649 cm^−1^ and 1599 cm^−1^ are attributed to the stretching vibration of C–O in the amide I (–NHCO–) and the N–H bending in the amide II (–NH_2_), respectively [42]. The bands at 1427 cm^−1^, 1383 cm^−1^, and 1323 cm^−1^ are assigned to the C−H bending vibration [45], and the bands at 1159 cm^−1^, 1061 cm^−1^, and 1031 cm^−1^ are corresponding to the C−O stretching vibration [46]. Compared to GO and CS, the spectrum of GOCS shows that a new band of amides appears at about 1659 cm^−1^ because the C−C and C=O bonds of GO interact with the –NH_2_ groups of CS to form the C=N bonds [28]. In addition, the broad bands at about 1383 cm^−1^ and 1061 cm^−1^ reflect the overlapping of the C–H vibration and C–O vibration of CS and GO at similar positions. For MGOCS, the band of amides is slightly shifted to the lower frequency of 1632 cm^−1^. Furthermore, the absorption bands at 895 cm^−1^ and 795 cm^−1^ are attributed to the O−H bending vibration, and the band at 572 cm^−1^ is attributed to the Fe−O lattice vibration [28]. After As(III) adsorption, the band of amides is slightly weakened, and the bands of the O−H and Fe−O groups significantly shift to the lower frequency, indicating that these groups are related to the MGOCS adsorption of As(III) and later may play a more important role.

#### 3.1.6. XPS Analysis

**Figure 6:**
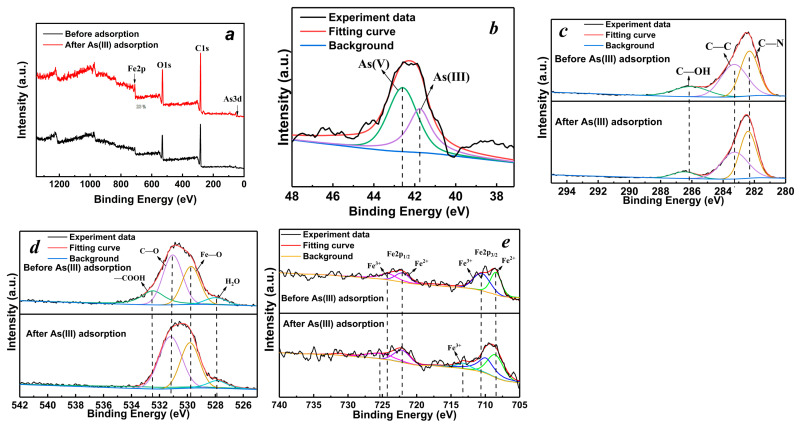
The XPS all-scan spectra of (**a**) MGOCS and XPS response of (**b**) As3d, (**c**) C1s, (**d**) O1s, and (**e**) Fe2p before and after As(III) adsorption.

The XPS scanning results of MGOCS before and after the adsorption of As(III) are shown in Figure 6. From the all-scan spectrums (Figure 6a), it can be seen that after adsorption, a new peak occurs at the binding energy of about 44 eV, which is attributed to the adsorbed As on the composite microspheres [47]. According to the spectra of As(3d), the adsorbed As is mainly composed of As(V) and As(III), and the distance between their peaks is about 9 eV, and the ratio of peak areas is about 1.5. This means that As(III) adsorption is accompanied by the partial oxidation of As(III) to As(V). No As(V) is detected in the aqueous solutions during adsorption; this is probably because As(III) in the aqueous solutions is adsorbed first and then the adsorbed As(III) is partially oxidized to As(V) on the MGOCS. The XPS spectra of Fe2p shows that the peaks at 708.45 eV (Fe 2p_3/2_) and 722.05 eV (Fe 2p_1/2_) are attributed to Fe(II), and the peaks at 710.53 eV(Fe 2p_3/2_) and 724.13 eV (Fe 2p_1/2_) are assigned to Fe(III). After adsorption, there is a significant shift and intensity decrease for the Fe(III) peak because Fe(III) is partially reduced to Fe(II). The spectra of C1s show that the peaks at 286.05 eV, 283.25 eV, and 282.32 eV correspond to C–OH, C–C, and C–N, respectively, and the spectra of O1s show that the peaks at 528.88 eV, 530.98 eV, and 531.58 eV correspond to Fe–O, –OH, and H_2_O, respectively. After As(III) adsorption, the peaks of C–OH, –OH, and Fe–O slightly shift to the aspect of higher binding energy, meaning that they are also involved in the adsorption reaction, which is consistent with the FTIR results in Section 3.1.6.

#### 3.1.7. SEM-EDS Analysis

**Figure 7:**
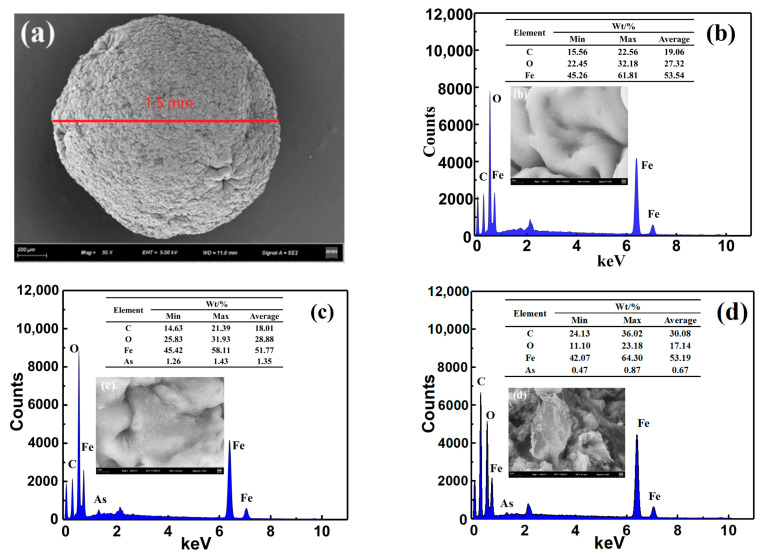
SEM images and EDS results of MGOCS composite microsphere before (**a**,**b**) and after (**c**,**d**) adsorption; the magnification of a is 50 and others are 5000; b, c show the surface image and elemental compositions; and d shows the interior result after cutting the microsphere.

The morphology analysis and EDS results of the MGOCS composite microspheres are shown in Figure 7. Compared to the smooth surface topography of the GOCS composite (Figure S3, Supplementary Materials) and the reported similar materials (stone-like morphology with the accumulation of grains existing as an irregular channel) [48,49], the surface of the MGOCS composites is rough, and there are a lot of folds (Figure 7a,b), meaning that iron oxide modification helps to provide a large surface area and enough adsorption sites for this composite to adsorb As(III). This can also be confirmed by the results in Section 3.1.1 and Section 3.2.1. The particle sizes of GOCS and MGOCS are about 1.5 mm. The major elements of GOCS are C (73.86%), O (22.25%), N, and others (3.91%) (Figure S3, Supplementary Materials), and the major elements of MGOCS are C (19.06%), O (27.32%), and Fe (53.54%) (Figure 7b). The high content of Fe for MGOCS and its slightly higher content of O than GOCS indicate that GOCS is modified well by iron oxide. After the MGOCS adsorption of As(III), the surface is covered by some flocs, and the major elements are C (18.01%), O (28.88%), Fe (51.77%), and As (1.35%) (Figure 7c). The occurrence of As and the decrease in Fe content (1.77%) indicate that the flocs correspond to the adsorbed As, and the adsorption is mainly related to the As reaction with iron oxides on the surface. Furthermore, the microsphere was cut and used to analyze the internal morphology and element composition. It was found that the internal structure appears to be granular and aggregated, and some flocs are also observed. The average content of As is 0.67% (Figure 7d), which is much less than the adsorbed As on the surface. This indicates that As can also be adsorbed into the internal particles of MGOCS through microporous channels, but the surface adsorption should still be the major adsorption mechanism.

### 3.2. Effect of Operational Parameters

#### 3.2.1. Fe Content

**Figure 8:**
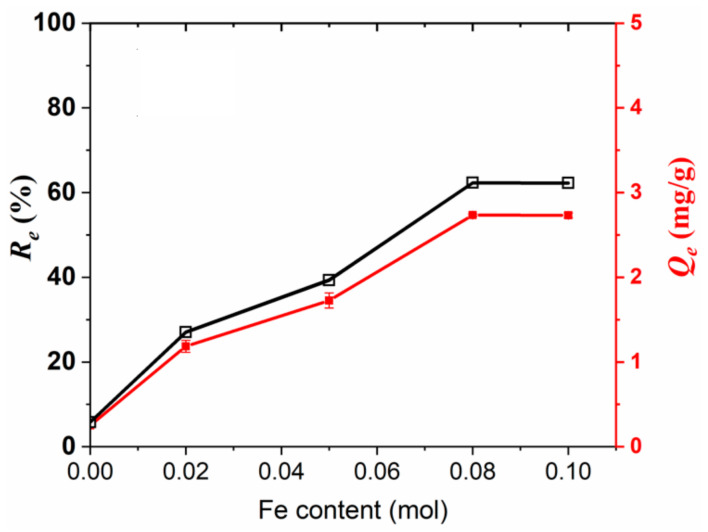
Changes in As(III) adsorption capacities (*Q_e_*) and removal efficiency (*R_e_*) with the Fe content.

Figure 8 shows the effect of the Fe content on the equilibrium adsorption capacity (*Q_e_*) and removal efficiency (*R_e_*) for 5 mg/L As(III) at 25 ± 1 °C. It can be seen that the values of *Q_e_* and *R_e_* are only 0.25 mg/g and 5.81% for GOCS, respectively. With the increase in Fe content from 0.02 mol to 0.08 mol, the value of *Q_e_* greatly increases from 1.19 mg/g to 2.73 mg/g, and the value of *R_e_* greatly increases from 27.04% to 62.30%. After that, *Q_e_* and *R_e_* remain stable. Thus, 0.08 mol FeCl_2_ is determined to be the optimum condition to prepare MGOCS. It is worth noting that the decorated Fe can be partially released from the composite of MGOCS, but the concentrations of Fe in the aqueous solutions are in the range of 0–0.05 mg/L during As(III) removal, which is much lower than the WHO drinking water limitation of 0.3 mg/L, indicating that MGOCS cannot cause Fe secondary pollution and FeCl_2_ treatment in this study is an eco-friendly magnetization decoration method.

#### 3.2.2. Mass and Volume Ratio (*m*/*v*)

**Figure 9:**
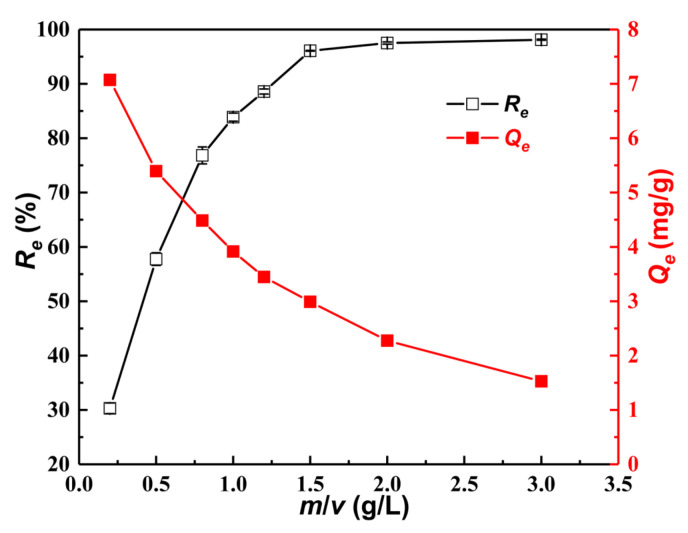
Changes in As(III) adsorption capacities (*Q_e_*) and removal efficiency (*R_e_*) with the *m/v*.

Figure 9 shows the changes in the equilibrium adsorption capacity (*Q_e_*) and removal efficiency (*R_e_*) of 5 mg/L As(III) under different values of *m*/*v* at 25 ± 1 °C. It can be seen that the value of *R_e_* increases with the increasing values of *m*/*v* because of the availability of sufficient adsorption sites on the surface of the Fe_3_O_4_–GOCS composite microspheres [50]. Specifically, *R_e_* quickly increases from 30.30% to 96.09% when the *m*/*v* value increases from 0.2 g/L to 1.5 g/L. After that, *R_e_* slowly increases to 98.13% when *m*/*v* increases to 3.0 g/L. However, *Q_e_* gradually decreases from 7.07 mg/g to 1.52 mg/g as the *m*/*v* value increases from 0.2 g/L to 3.0 g/L. To obtain a higher *R_e_* and ensure a sufficient adsorption capacity, the optimal *m*/*v* is determined to be about 1.0 g/L.

#### 3.2.3. Effect of Initial As(III) Concentration

**Figure 10:**
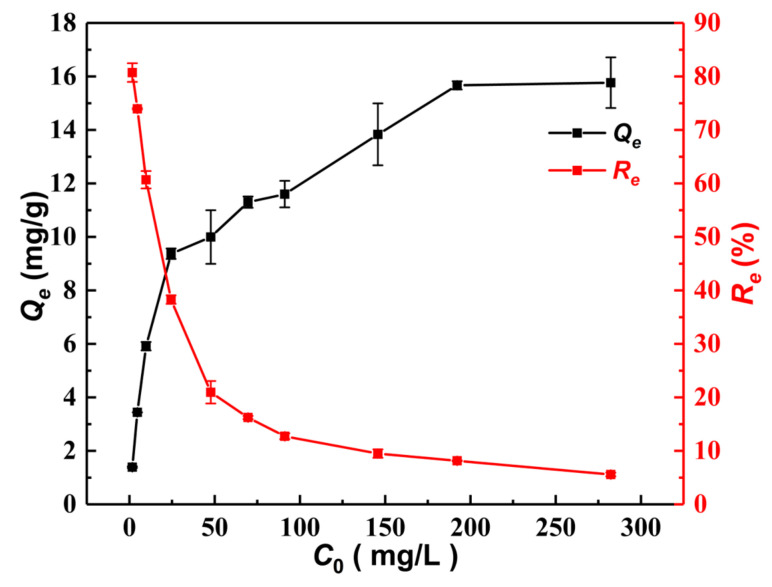
Changes in As(III) adsorption capacities (*Q_e_*) and removal efficiency (*R_e_*) with the initial concentration of As(III).

Figure 10 shows the effect of initial As(III) concentrations (*C*_0_) on the equilibrium adsorption capacity (*Q_e_*) and removal efficiency (*R_e_*) at 25 ± 1 °C. It can be seen that with the increase in *C*_0_ from 2 mg/L to 200 mg/L, *Q_e_* gradually increases from 1.34 mg/L to 15.67 mg/L and keeps stable afterward. However, the value of *R_e_* rapidly decreases from 80.72% to 20.95% with the increasing *C*_0_ from 2 mg/L to 50 mg/L. Then, *R_e_* slowly decreases to the lowest value of 5.59% when *C*_0_ rises to 280 mg/L. Overall, with the increasing *C*_0_, the value of *Q_e_* shows an increasing trend until keeping stable, while *R_e_* tends to decrease until it is stable. Similar results have been found for As(III) adsorption by many different types of composites, such as polyaniline hollow microsphere magnetic nanocomposites (PNHM/Fe_3_O_4_) [51], α–FeO(OH)/GO/CS [12], and copper-coated MnO_2_ nanowire membranes [52]. This may be because As(III) adsorption by MGOCS mainly occurs at the solid–liquid interface and depends on the active sites and pores of the functional groups on the solid surface [12].

#### 3.2.4. Initial pH

**Figure 11:**
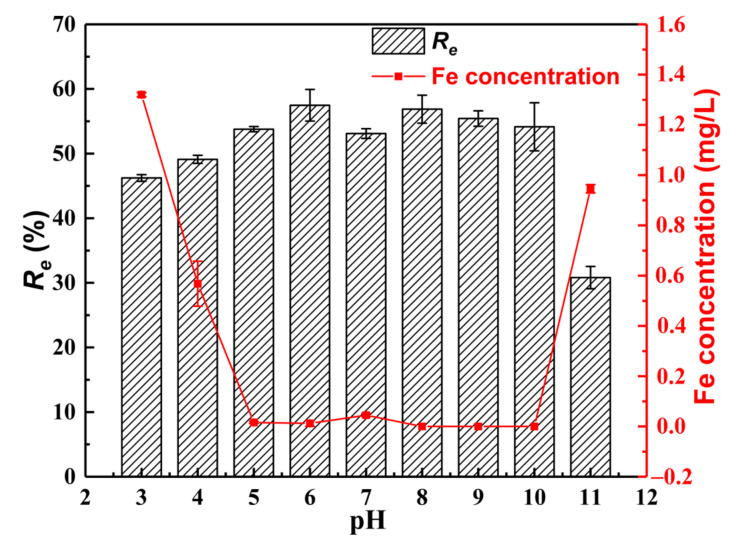
Changes in As(III) adsorption capacities (*Q_e_*) and removal efficiency (*R_e_*) as well as the concentrations of Fe in an aqueous solution after As(III) adsorption under varying pH.

Figure 11 shows the removal efficiency (*R_e_*) of 5 mg/L As(III) under varying pH as well as the concentrations of Fe in an aqueous solution after As(III) adsorption. It is shown that *R_e_* increases from 46.24% to 57.48% with the increasing pH from 3 to 6. When the pH is 7, *R_e_* is 53.09%. With the increasing pH from 8 to 11, *R_e_* decreases from 56.87% to 30.81%. Over a wide pH range of 5–10, the concentrations of Fe in the aqueous solutions are in the range of 0–0.05 mg/L after As(III) adsorption, far below the WHO drinking water limit of 0.3 mg/L. Meanwhile, MGOCS shows a high *R_e_* of As(III) (>53%). To obtain the best removal efficiency, a pH of 6 is determined to be the optimal condition for As(III) removal.

The pH value at the point of zero charges (pH*_pzc_*) for MGOCS is 9.46 (Figure S4, Supplementary Materials). This means that the surface of the MGOCS is positively charged (caused by protonation) when the pH value is below 9.46 and is negatively charged when the pH value is higher than 9.46. Furthermore, As (III) mainly exists as the neutral form (H_3_AsO_3_) in the aqueous phase below pH 9.2 and the anionic (H_2_AsO_3_^−^) form in the pH range of 9.2–12.1 [53,54]. Therefore, MGOCS can adsorb a great number of As(III) from an aqueous solution through surface complexation and electrostatic attraction when the pH value is below 10. However, the electrostatic repulsion can cause a great decrease in As(III) adsorption when the pH value increases from 10 to 11. In addition, the XPS analysis in Section 3.1.6 shows that the adsorbed As(III) can be partially oxidized to As(V), which mainly exists as H_2_AsO_4_^−^ at a pH less than 6.69 and HAsO_4_^2−^ at a pH of 6.69–11.5 [53]. Therefore, when the aqueous pH is less than 6, As(III) is mainly adsorbed through surface complexation at first, and then it is partially oxidized to As(V) which will be adsorbed through electrostatic attraction between H_2_AsO_4_^−^ and the adsorbent, further enhancing the As(III) removal. Meanwhile, OH^−^ is released to the aqueous solutions from the composite through ion exchange with H_2_AsO_4_^−^; thus, the final pH values will increase after adsorption (Figure S5, Supplementary Materials). With the aqueous pH increasing from 6 to 10, the decreasing trend of the As(III) removal efficiency may be attributed to the fact that an increasing amount of OH^−^ can compete for more adsorption sites and exchange with the adsorbed As(III) (in the form of H_2_AsO_3_^−^) and As(V) (in the form of HAsO_4_^2−^). This can be further confirmed by the result that the final pH value decreases after adsorption (Figure S5, Supplementary Materials).

#### 3.2.5. Coexisting Ions

**Figure 12:**
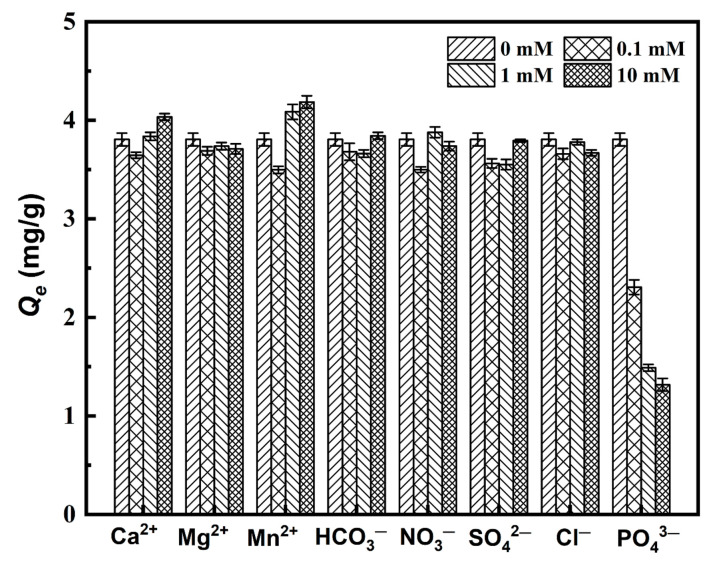
Effects of coexisting ions on the equilibrium adsorption capacity (*Q_e_*) of As(III).

Various anions and cations in natural water may interfere with As(III) adsorption through competitive binding or complexation. Figure 12 shows the effects of adding different ions on the equilibrium adsorption capacity (*Q_e_*) for 5 mg/L As(III) at 25 °C. It can be seen that the added cations of Ca^2+^ and Mg^2+^ and the anions of HCO_3_^−^, SO_4_^2−^, and NO_3_^−^ show fewer effects on MGOCS adsorption of As(III). Different from these ions, the added PO_4_^3−^ plays a significantly inhibitory role in the adsorption of As(III), and this effect becomes stronger with increasing concentration. It is found that *Q_e_* gradually decreases from 3.81 mg/g to 1.32 mg/g when the added PO_4_^3−^ increases from 0 to 10 mmol. This may be attributed to the fact that PO_4_^3−^ has a stronger affinity for iron oxides than As(III) and easily forms surface complexes with hydroxyl groups within the aspheric surface [55,56] and can greatly compete for adsorption sites with the As(V) produced by the adsorbed As(III) oxidization. In addition, the presence of a higher concentration of Mn^2+^ can slightly enhance the adsorption of As(III). *Q_e_* increases from 3.50 mg/g to 4.19 mg/g when the added Mn^2+^ increases from 0.1 to 10 mM. This may be attributed to the fact that Mn^2+^ can also be adsorbed onto the surface of MGOCS, which enhances the electrostatic force between the adsorbent and As and thus facilitates As adsorption [57].

### 3.3. Adsorption Characteristics

#### 3.3.1. Adsorption Kinetics

**Figure 13:**
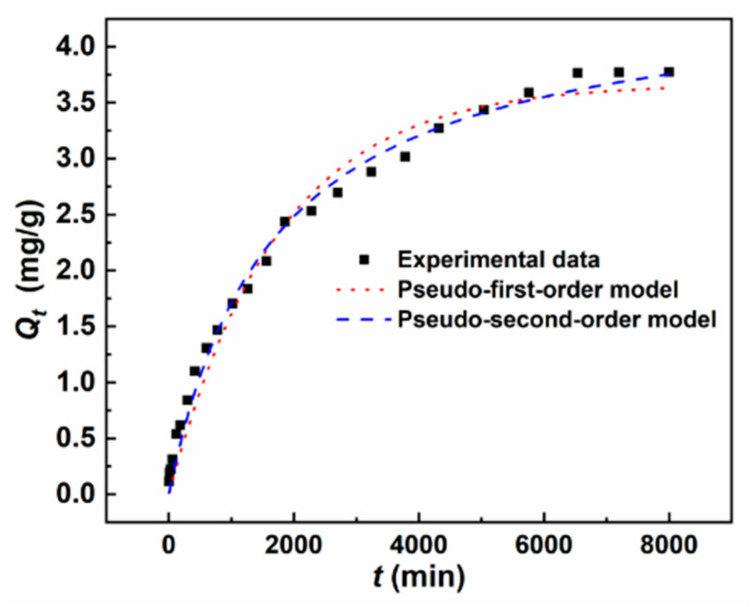
The best-fitted curves of As(III) adsorption by MGOCS using pseudo-first-order and pseudo-second-order kinetic models, respectively.

Figure 13 represents the changes in the adsorption capacity (*Q_t_*) of As(III) with the contact time. With the increase in contact time, *Q_t_* gradually increases before 6540 min and reaches equilibrium after that. This can be explained by the fact that in the initial stage, the adsorbent surface has enough available active adsorption sites, and the concentration difference between the aqueous and adsorbed phases is strong enough to keep a fast rate of As(III) adsorption. As the reaction proceeds, the available adsorption sites decrease, and the concentration difference between the aqueous and adsorbed phases drops, resulting in a decrease in the adsorption rate [12]. To further investigate the adsorption process, we have used the pseudo-first-order and pseudo-second-order kinetic models to fit the experimental data and the best-fitting results are shown in Figure 13 and Table 1. It can be seen that the experimental data are better fitted to the pseudo-second-order kinetic than the pseudo-first-order kinetic, and their determination coefficients (*R*^2^) are 0.99 and 0.97, respectively. This implies that the MGOCS sorption of As(III) is mainly conducted by forming a monolayer on the surface via chemisorption, and the migration process is controlled by a second-order rate equation [21,58]. Combining with the results from SEM-EDS in Section 3.1.7, the MGOCS adsorption of As(III) can be divided into three steps. Firstly, there is rapid adsorption of As(III) on the adsorbent surface. Then, As(III) slowly diffuses into the interior of the adsorbent through the pore throats. Finally, the MGOCS adsorption of As(III) reaches equilibrium.

#### 3.3.2. Adsorption Isotherms

**Figure 14:**
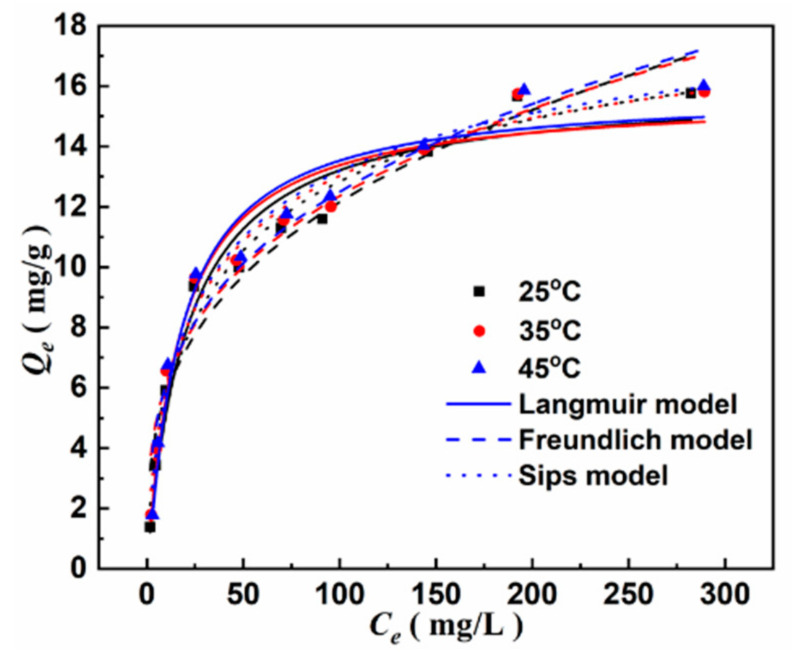
Adsorption isotherm fitting curves of As(III) on MGOCS composite.

Figure 14 shows the isothermal adsorption of As(III) under different temperatures of 25 °C, 35 °C, and 45 °C, respectively. It can be seen that the equilibrium adsorption capacity (*Q_e_*) of As(III) gradually increases with the increase in As(III) equilibrium concentration (*C_e_*) and finally tends to be stable with *C_e_* larger than 200 mg/L, indicating that MGOCS exhibits better adsorption of higher concentrations of As(III). The values of *Q_e_* for the same concentration of As(III) adsorption are similar under different temperatures, meaning that temperature hardly affects the MGOCS adsorption of As(III). Using the Langmuir, Freundlich, and Sips models to fit the experimental data, the best-fitted curves and parameters are shown in Figure 14 and Table 2, respectively. It can be seen that experimental data of As(III) adsorptions fit well with these three models, but the coefficient of the determination (*R*^2^) obtained by the Sips model (0.97) is larger than those of the Langmuir (0.95) and Freundlich (0.94) models. This means that a low concentration of As(III) sorption isotherm likely follows the Freundlich isotherm model, while a high concentration of As(III) sorption isotherm likely follows the Langmuir isotherm model [59]. The Langmuir model assumes that adsorption occurs on a homogeneous surface and that all binding sites have the same affinity for the adsorbent, with predominantly monolayer adsorption [60,61]. The Freundlich model assumes that the adsorbent has a heterogeneous surface structure and considers multilayer adsorption as the dominant process [62]. Therefore, MGOCS adsorption of the low concentrations of As(III) is mainly heterogeneous multilayer adsorption, and the maximum adsorption capacity (*Q_m_*) is mainly determined by the monolayer adsorption, which is 20.72 mg/g at a pH of 6 and 25 °C as fitted by the Sips model.

### 3.4. Regeneration and Performances Evaluation

**Figure 15:**
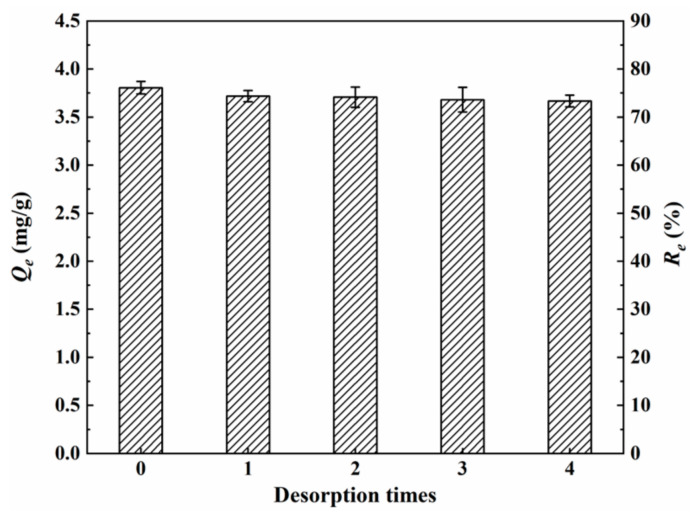
Changes in the removal efficiency (*R_e_*) of MGOCS over four times of regeneration.

The regeneration and reusability of the adsorbents are important factors for their practical applications [19]. Figure 15 shows the equilibrium adsorption capacity (*Q_e_*) and removal efficiency of As(III) after desorption using 0.1 M NaOH. It can be seen that the values of *Q_e_* and *R_e_* show slight decreasing trends after each desorption. After four times of desorption, *Q_e_* decreases from 3.81 mg/g to 3.67 mg/g, and *R_e_* decreases from 76.2% to 73.4%, implying that the prepared MGOCS in this work has good reusability.

Table 3 compares the MGOCS composite with other similar magnetic, GO-based, or CS-based composite materials for As(III) removal. It can be seen that the MGOCS prepared in this study has some advantages. Most adsorbents can only efficiently remove As(III) in a neutral or weak alkaline environment and may become unstable or show low adsorption capacity for As(III) in acidic environments [19,63,64]. The prepared MGOCS in this work can efficiently adsorb As(III) over the wide pH ranges of 5–10, and this process is almost not affected by reaction temperatures. The maximum adsorption capacity of As(III) (*Q_m_*) is 20.72 mg/g at a pH of 6, 25 °C, which is higher than that of similar composites, such as α-Fe_2_O_3_-impregnated CS beads, FeOOH-CS beads, etc. In addition, most reported methods usually use Fe(III) or a certain ratio of Fe(III) and Fe(II) to react at a high temperature to create Fe_3_O_4_ and magnetic decoration for the composite [19,23,31,33,34], and such complicated processes easily lead to the Fe_3_O_4_ speciation, stability, and magnetic force not being satisfactory. In this work, magnetite decoration is easily operated by using Fe(II) to react with the GOCS mixtures at 45 °C, and the obtained MGOCS (with a particle size of 1.5 mm) shows a large surface area of 67.39 m^2^/g, a good magnetization intensity of 24.35 emu/g, and strong thermal stability. It is worth noticing that MGOCS is eco-friendly and will not cause secondary pollution of Fe during its application for As(III) removal. When MGOCS is used to adsorb 10 mg/L Cr(VI), Cu(II), Pb(II), Cd(II), and As(V) at a pH of 6.0, *m/v* of 1 g/L and 25 °C, the removal efficiency of Cr(VI) and As(V) exceeds 60% and that of Cu(II), Cd(II), and Pb(II) are 6.60%, 11.35%, and 9.09%, respectively (Figure S6, Supplementary Materials). This indicates that MGOCS also has a good application prospect for Cu(II), Cd(II), and Pb(II) removal, especially for Cr(VI) and As(V) removal from an acidic aqueous environment. In addition, the actual wastewater containing As(III) and other metals are usually complicated and various; therefore, the performance of MGOCS applied for wastewater treatment needs further study in the future.

### 3.5. Adsorption Mechanism

Chemosorption is the major reaction mechanism for As(III) removal, which is mainly related to the functional groups of Fe–O, –OH, and amides of the composite and is accompanied by intraparticle diffusion. Specifically, As(III) (in the form of H_3_AsO_3_) is firstly adsorbed on the surface of MGOCS through surface complexation, and then the adsorbed As(III) is partially oxidized to As(V) accompanied by the reduction of Fe(III) to Fe(II). On the one hand, the produced As(V) can be further adsorbed by the MGOCS through ligand exchange to form single-tooth mononuclear or double-tooth binuclear Fe–O–As complexes. On the other hand, the anionic As(III) in the form of H_2_AsO_3_^−^ and As(V) in the form of H_2_AsO_4_^−^ can be adsorbed through electrostatic attraction because the surface of MGOCS is protonated and positively charged at a pH of <9.46. These will further enhance As(III) removal from an aqueous solution. The major reactions are as follows.

(8) $$\mathrm{Fe}\left(\mathrm{II}\right)/\mathrm{Fe}\left(\mathrm{III}\right)-O+\mathrm{As}\left(\mathrm{III}\right)\to \;\mathrm{Fe}\left(\mathrm{II}\right)/\mathrm{Fe}\left(\mathrm{III}\right)-O-\mathrm{As}\left(\mathrm{III}\right)\;\mathrm{complex}$$

(9) $$\mathrm{Fe}\left(\mathrm{III}\right)-O+\mathrm{As}\left(\mathrm{III}\right)\;\to \;\mathrm{Fe}\left(\mathrm{II}\right)-O-\mathrm{As}\left(V\right)\;\mathrm{complex}$$

(10) $$R-\mathrm{OH}+{\;H}^{+}\;\to \;R-{\mathrm{OH}}_{2}^{+}$$

(11) $$R-{\mathrm{OH}}_{2}^{+}+H_{2}{\mathrm{As}}^{\mathrm{III}}O_{3}^{-}\;\to R-H_{2}{\mathrm{As}}^{\mathrm{III}}O_{3}+{\;H}_{2}O$$

(12) $$R-{\mathrm{OH}}_{2}^{+}+H_{2}{\mathrm{As}}^{V}O_{4}^{-}\;\to R-H_{2}{\mathrm{As}}^{V}O_{4}+{\;H}_{2}O$$

(13) $$R-{\mathrm{NH}}_{3}^{+}+{\;H}_{2}{\mathrm{As}}^{\mathrm{III}}O_{3}^{-}\;\to \;R-{\mathrm{NH}}_{3}^{+}\cdots H_{2}{\mathrm{As}}^{\mathrm{III}}O_{3}^{-}$$

(14) $$R-{\mathrm{NH}}_{3}^{+}+{\;H}_{2}{\mathrm{As}}^{V}O_{4}^{-}\;\to \;R-{\mathrm{NH}}_{3}^{+}\cdots H_{2}{\mathrm{As}}^{V}O_{4}^{-}$$

## 4. Conclusions

In this work, the MGOCS composite microsphere was specifically synthesized and used for the efficient removal of As(III) from aqueous solutions. The major conclusions are as follows:

(1) Using Fe(II) to react with GOCS mixtures at 45 °C is an easily operational magnetic decoration method, and the obtained MGOCS composite shows a large surface area of 67.39 m^2^/g, a good magnetization intensity of 24.35 emu/g, strong thermal stability, and is a type of eco-friendly composite for highly efficient As(III) removal.
(2) MGOCS can efficiently adsorb As(III) over a wide pH range of 5–10. The adsorption capacity increases with the increasing values of Fe content and initial As(III) concentration and decreases with the increasing *m*/*v* but is almost unaffected by temperature. The coexisting ion of PO_4_^3−^ can greatly weaken As(III) adsorption, while Mn^2+^ can slightly enhance As(III) adsorption. After four cycles of regeneration, As(III) removal efficiency only decreases by 2.8%, indicating that the composites can be recycled and reused well.
(3) As(III) kinetic adsorption on MGOCS is best fitted to the pseudo-second-order kinetic model with a coefficient of determination (*R*^2^) greater than 0.99. MGOCS adsorption of a low concentration of As(III) is mainly heterogeneous multilayer adsorption, and the maximum adsorption capacity is determined by the monolayer adsorption, which is 20.72 mg/g at a pH of 6 and 25 °C as fitted by the Sips model.
(4) As(III) removal by MGOCS is related to the Fe–O, O–H, and amides groups. It is first adsorbed on the surface and the interior of the MGOCS attraction, and then the adsorbed As(III) can be partially oxidized to As(V) by Fe(III) reduction to Fe(II), and the produced As(V) can be greatly adsorbed by the composite through complexation and electronic attraction, further enhancing the As(III) removal.

## Figures and Tables

**Table 1:** Fitting parameters of pseudo-first-order and second-order kinetics for 5 mg/L As(III) adsorption on MGOCS.

Models	Time (min)	Parameters		
Pseudo-first-order kinetic	0–8000	*Q_e_* (mg/g)	*k*_1_ (min^−1^)	*R* ^2^
		3.67	0.00058	0.9792
Pseudo-second-order kinetic		*Q_e_* (mg/g)	*k_2_* (mg (g·min) ^−1^)	*R* ^2^
		4.52	0.00013	0.9906

**Table 2:** Isotherm parameters of As(III) adsorption on MGOCS.

Temperature (°C)	Langmuir Model			Freundlich Model			Sips Model			
	*Q_m_* (mg/g)	*K_L_*	*R* ^2^	*K_f_*	1*/n*	*R* ^2^	*Q_m_* (mg/g)	*K_s_*	1*/m*	*R* ^2^
25	15.97	0.0478	0.9581	2.75	0.3231	0.9491	20.72	0.0802	0.65	0.9748
35	15.70	0.0572	0.9546	3.06	0.3029	0.9465	20.31	0.0950	0.64	0.9743
45	15.78	0.0579	0.9558	3.09	0.3025	0.9461	20.28	0.0952	0.64	0.9749

**Table 3:** Comparison of As(III) removal by MGOCS and other similar composites.

Adsorbents	pH	T( °C)	As(III) (mg/L)	*m*/*v* (g/L)	*Q_m_* (mg/g)	Reusability	Ref.
GO with 3-aminopyrazole	8.3	25	10~60	0.33	131.6	3 times, 88.64%	[65]
GO	7.0	23	25~1200	0.8	19	―	[66]
GO with different degrees of oxidation	7.0	25	1~700	1.25	123~288	―	[67]
Fe-CS	7.0	25	1~10	5.0	16.2	2 times, stable	[68]
Nanoscale zero-valent iron-reduced GO	7.0	25	1~15	0.4	35.8	―	[69]
Fe_3_O_4_/GO/CS	7.3	25	10	5.0	45.5	5 times, 47.7%	[19]
α-Fe_2_O_3_-impregnated CS beads	5.0	30	10~100	5.0	9.4	10 times, lost 20.2%	[63]
FeOOH-CS beads	6.5	25±1	1~50	3.3	7.24	―	[70]
MGOCS	6.0	25	2–200	1.0	20.72	4 times, >70%	This work

## Data Availability

All data used during the study appear in the submitted article.

## Supplementary Materials

The following supporting information can be downloaded at https://www.mdpi.com/article/10.3390/ma15207156/s1: Figure S1: Photos of MGOCS composite microsphere; Figure S2: BET analysis of the GOCS: (a) adsorption/desorption isotherm plots, (b) bar graphs of the BET and BJH surface areas, and (c) BJH plots of the pore size distribution; Figure S3: SEM images and EDS results of GOCS composite microsphere. Figure S4: The changes in the ζ potential of adsorbent with the pH values; Figure S5: Variations of pH values after As(III) adsorption; Figure S6: Removal efficiency (Re) of MGOCS for different heavy metals.
