# Supplementary material for: On As(III) Adsorption Characteristics of Innovative Magnetite Graphene Oxide Chitosan Microsphere
Source: Materials (Basel). 2022 Oct 14;15(20):7156. doi: 10.3390/ma15207156 (PMC9605594; doi:10.3390/ma15207156)
Supplement: Supplementary file 1 [file materials-15-07156-s001.zip › materials-1953698-supplementary.pdf]

Supporting Materials

On As(III) Adsorption Characteristics of Innovative Magnetite Graphene Oxide Chitosan Microsphere

Huimei Shan [1,2], Yunquan Liu [1,2], Chunya Zeng [1,2], Sanxi Peng [1,*] and Hongbin Zhan [3,*]

[1] Guangxi Key Laboratory of Environmental Pollution Control Theory and Technology, Guilin University of Technology, Guilin 541004, China
[2] College of Environmental Science and Engineering, Guilin University of Technology, Guilin 541004, China
[3] Department of Geology & Geophysics, Texas A&M University, College Station, TX 77843, USA
* Correspondence: pengsanxi1984@163.com (S.P.); zhan@tamu.edu (H.Z.); Tel.: +1-(979)-862-7961 (H.Z.); Fax: +1-(979)-845-6162 (H.Z.)

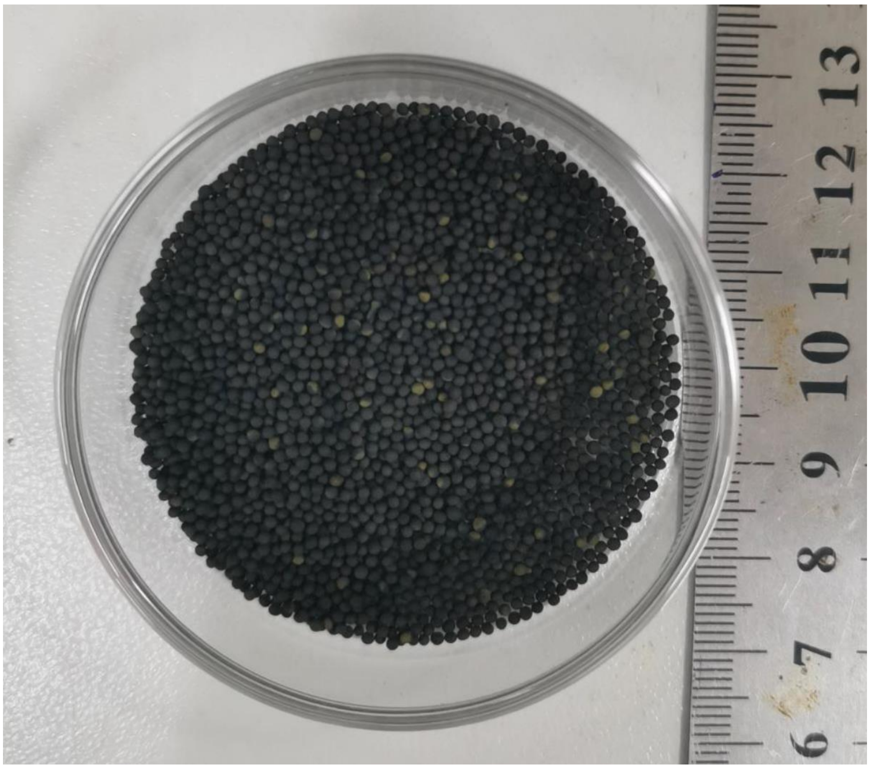

Figure S1. Photos of MGOCS composite microsphere.

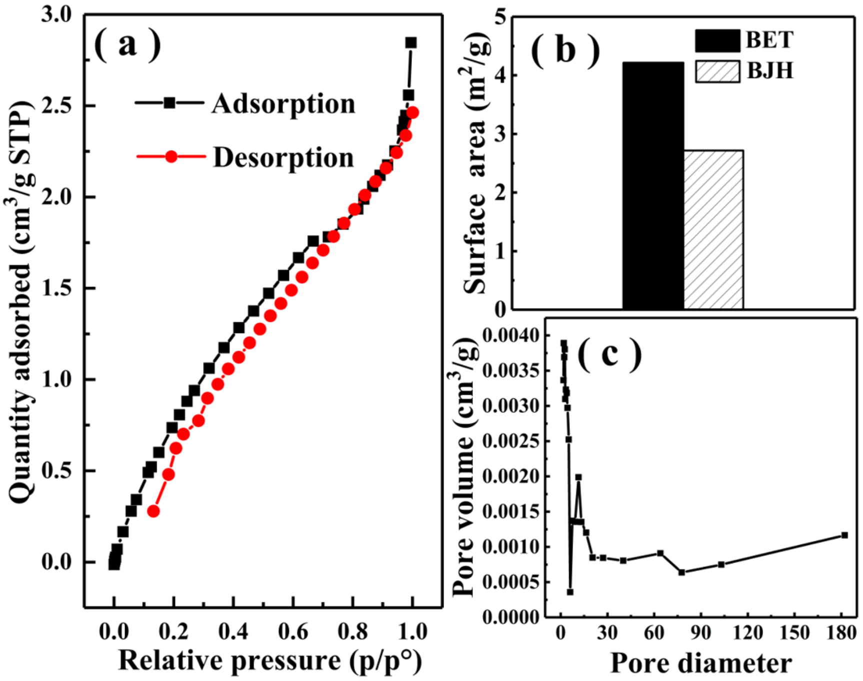

Figure S2. BET analysis of the GOCS: (a) adsorption/desorption isotherm plots, (b) bar graphs of the BET and BJH surface areas, and (c) BJH plots of the pore size distribution.

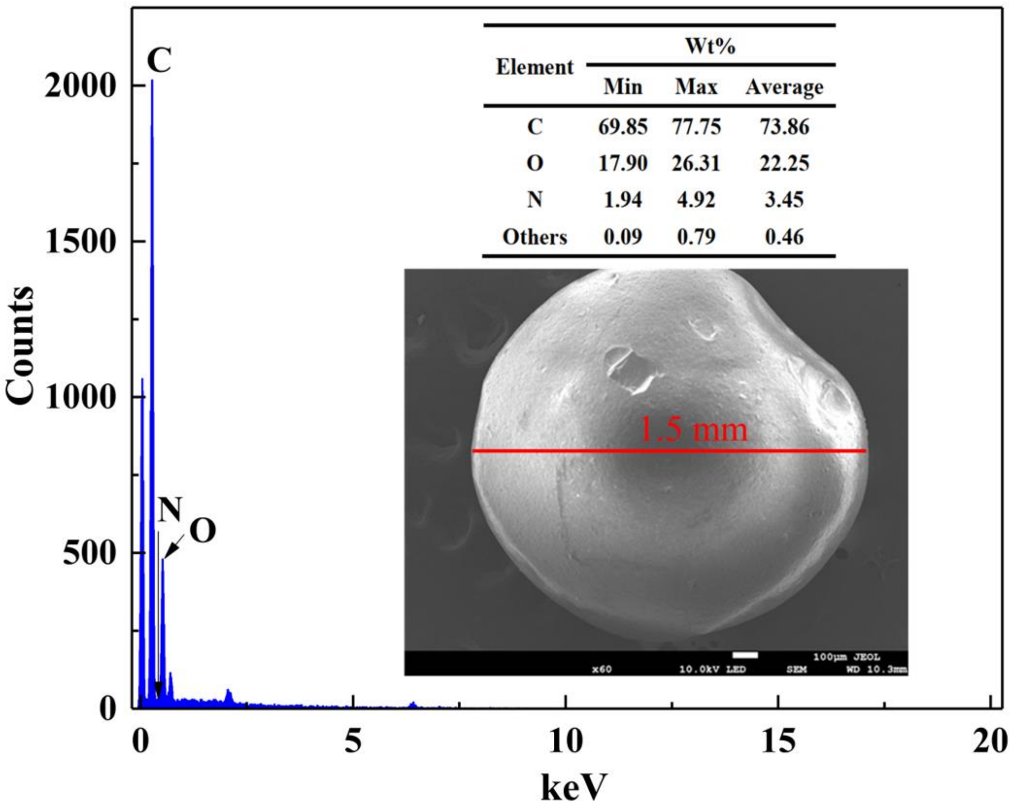

Figure S3. SEM images and EDS results of GOCS composite microsphere.

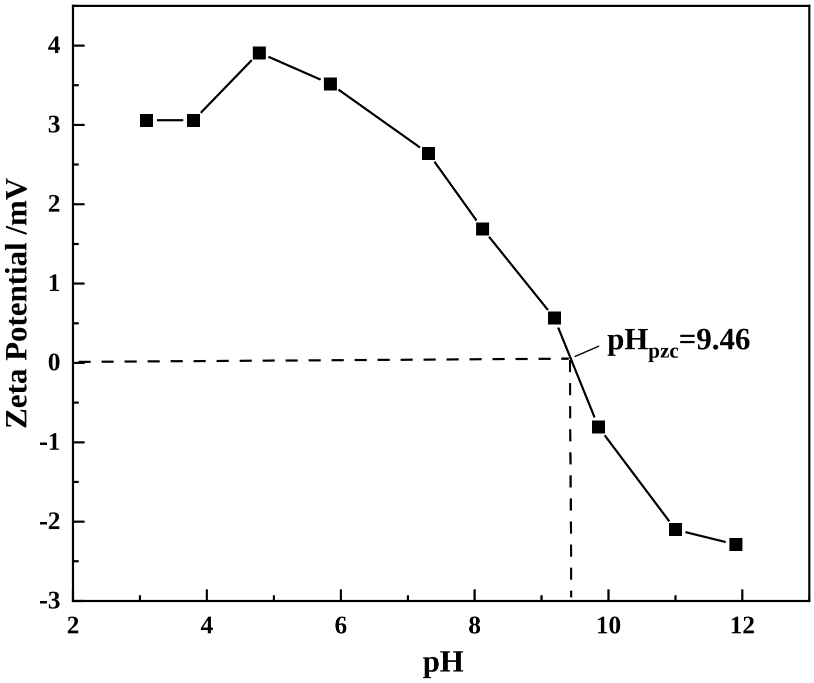

Figure S4. The changes in the ζ potential of adsorbent with the pH values.

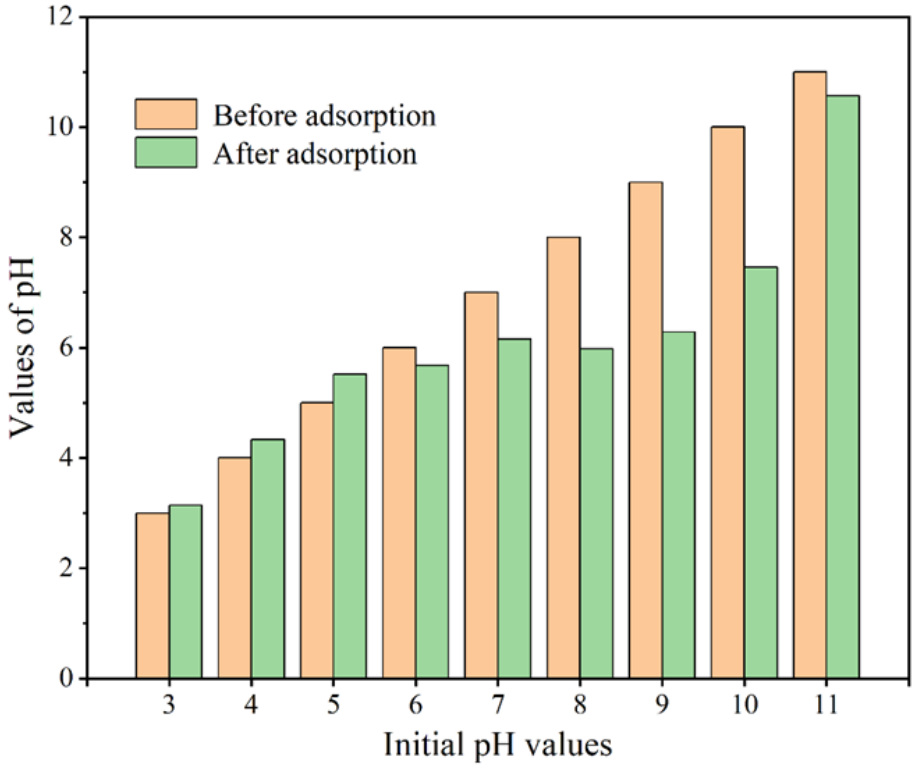

Figure S5. Variations of pH values after As(III) adsorption.

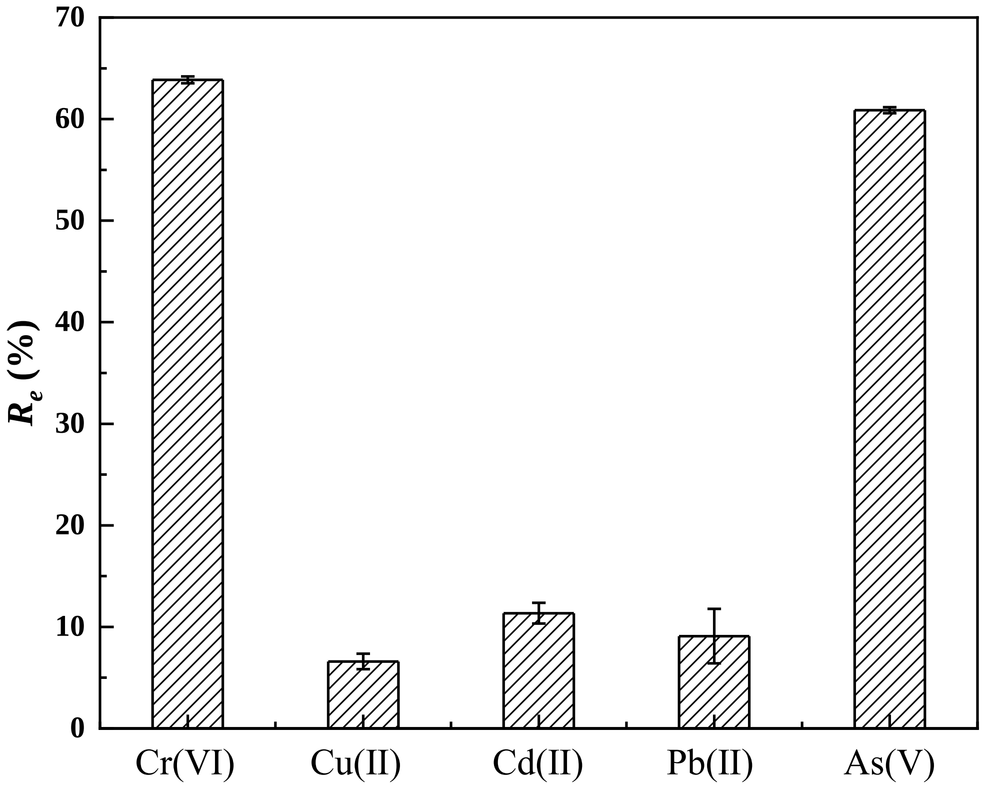

Figure S6. Removal efficiency (R_e) of MGOCS for different heavy metals.
